# Serotonin Transporter Genotype Modulates Social Reward and Punishment in Rhesus Macaques

**DOI:** 10.1371/journal.pone.0004156

**Published:** 2009-01-14

**Authors:** Karli K. Watson, Jason H. Ghodasra, Michael L. Platt

**Affiliations:** 1 Department of Neurobiology, Duke University, LSRC Room, Durham, North Carolina, United States of America; 2 Feinberg School of Medicine, Northwestern University, Chicago, Illinois, United States of America; Yale University, United States of America

## Abstract

**Background:**

Serotonin signaling influences social behavior in both human and nonhuman primates. In humans, variation upstream of the promoter region of the serotonin transporter gene (5-HTTLPR) has recently been shown to influence both behavioral measures of social anxiety and amygdala response to social threats. Here we show that length polymorphisms in 5-HTTLPR predict social reward and punishment in rhesus macaques, a species in which 5-HTTLPR variation is analogous to that of humans.

**Methodology/Principal Findings:**

In contrast to monkeys with two copies of the long allele (L/L), monkeys with one copy of the short allele of this gene (S/L) spent less time gazing at face than non-face images, less time looking in the eye region of faces, and had larger pupil diameters when gazing at photos of a high versus low status male macaques. Moreover, in a novel primed gambling task, presentation of photos of high status male macaques promoted risk-aversion in S/L monkeys but promoted risk-seeking in L/L monkeys. Finally, as measured by a “pay-per-view” task, S/L monkeys required juice payment to view photos of high status males, whereas L/L monkeys sacrificed fluid to see the same photos.

**Conclusions/Significance:**

These data indicate that genetic variation in serotonin function contributes to social reward and punishment in rhesus macaques, and thus shapes social behavior in humans and rhesus macaques alike.

## Introduction

The synaptic serotonin transporter plays a crucial role in regulating emotion in both human and non-human primates. Expression levels of the serotonin transporter gene depend on the serotonin transporter linked polymorphic region (5-HTTLPR), a sequence of tandem repeats upstream of the promoter that is polymorphic in humans and simian primates [1]. Humans and rhesus macaques have short (S) and long (L) allelic variants of 5-HTTLPR, and in both species the presence of the S allele interacts with early environment to produce long term effects on behavior, personality, and measures of central nervous system function [2]–[5]. Presence of the S allele in captive rhesus macaques predisposes them towards increased alcohol consumption [6], exacerbated neuroendocrine responses to stress [3], and greater rates of affective responding [4]. Similarly, human S carriers who experience childhood abuse or trauma are at elevated risk of alcoholism and depression [2]. Moreover, functional imaging studies indicate that human S carriers exhibit enhanced amygdala response to social threats such as angry faces [7], [8].

Based on these observations, we predicted that allelic variation in 5-HTTPLR would influence individual reactivity to social reward and punishment in rhesus macaques, as it appears to do in humans. We tested this hypothesis in three complimentary experiments: First, we measured eye gaze patterns and pupil diameter in male rhesus macaques when they were given the opportunity to look at images of other rhesus macaques; second, we measured the effects of seeing social images on subsequent gambling for juice rewards; and third, we measured the amount of juice male rhesus macaques sacrificed or demanded for the opportunity to see these images. These experiments provide three implicit measures of the influence of social stimuli on neural systems mediating reward and punishment [9]–[11].

## Results

### 5-HTTLPR genotype modulates gaze pattern and pupil diameter in rhesus macaques when viewing social images

**Figure 1:**
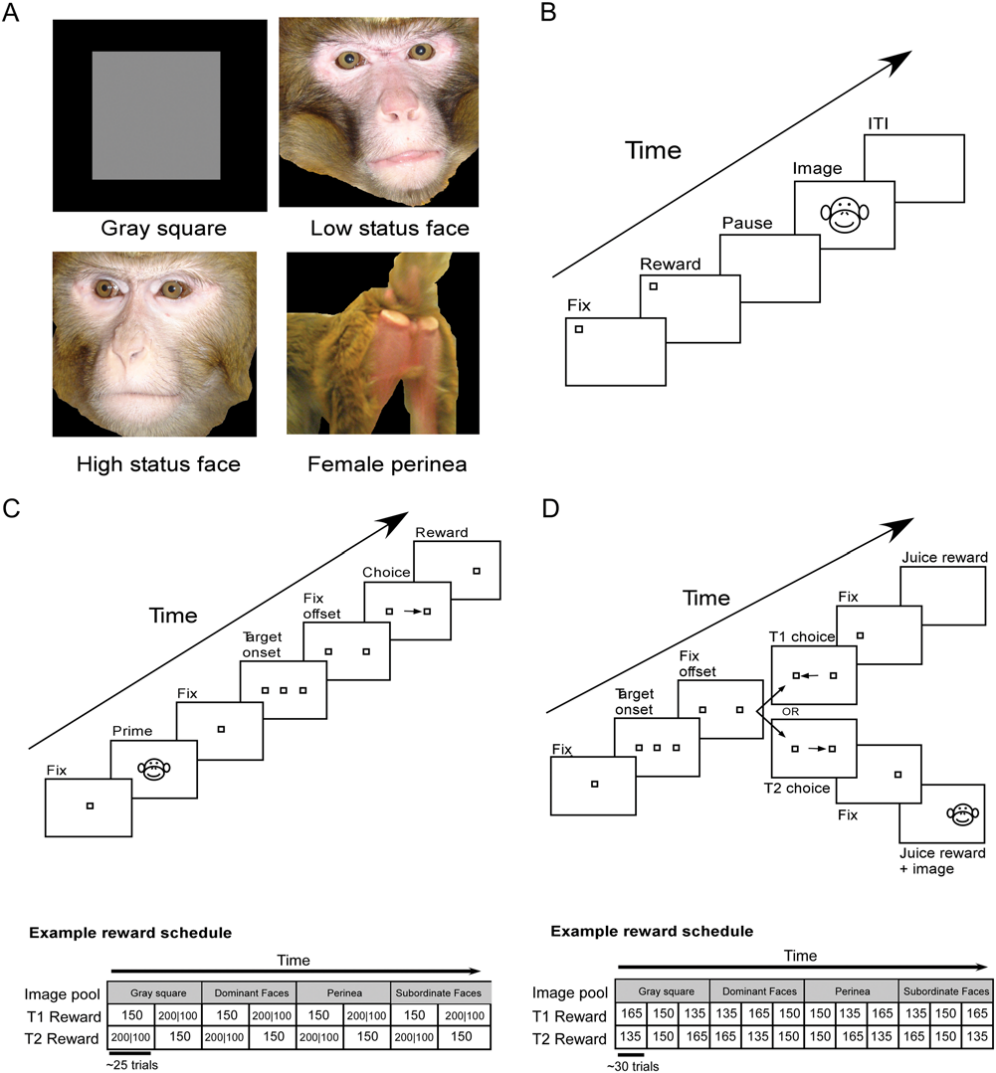
Tasks used to assess the influence of 5-HTTLPR genotype on social reward and punishment. (A) Stimuli consisted of images of familiar conspecifics. Image pools used in the pay-per-view and primed risk taking task were identical, and consisted of four categories: gray square, faces of familiar low status individuals, faces of familiar high status individuals, and perinea of familiar females. Each of the three latter image pools consisted of 60 different images of either three (face pools) or four (perinea pool) different individuals. Images used for the free viewing task consisted of high and low status faces similar, but not identical, to those used in the other two tasks; and scrambled faces. Trial structures and reward schedules for (B) the free viewing task, (C) primed risk taking task, and (D) pay-per-view task. Stimuli for the free viewing task were randomly interleaved. The risk taking and pay-per-view tasks utilized a blocked trial structure so that reward contingencies were apparent to the animal after sampling each option.

**Figure 2:**
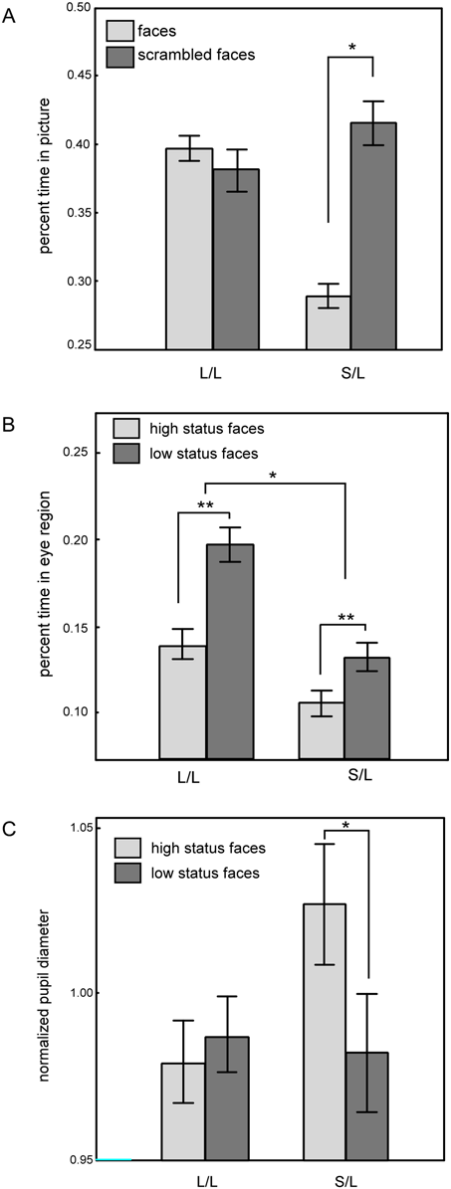
Serotonin transporter genotype influences eye position and pupil diameter when observing social images. (A) In contrast to L/L monkeys, S/L monkeys looked less at the picture when the image depicted a face than when it depicted a scrambled face (* indicates p = 0.02) and (B) spent less time looking in the eye region when face images were presented. Additionally, both L/L and S/L monkeys spent less time looking in the eye region of high status faces than low status faces (* indicates p = 0.02, ** indicates p = 0.01). (C) When observing faces, the pupil diameter of S/L monkeys was modulated by the social status of the displayed face, with a greater mean pupil diameter induced by the presentation of high-status faces (* indicates p = 0.05). In contrast, no significant difference in the pupil diameters of L/L animals was observed to correlate with image category.

Eight adult male rhesus macaques (four L/L and four S/L) were presented with a series of images depicting faces (see Figure 1A) or scrambled faces of familiar macaque monkeys (see Figure 1B for task sequence). Eye position and pupil diameter were monitored using an infrared camera based eye tracking system. S/L monkeys spent less total time looking at face images relative to scrambled face images (27.9±7.7% for face images, 40.5±11.0% for scrambled), whereas L/L monkeys looked equally at both image categories (39.8±12.9 face versus 38.1±16.9 scrambled; Repeated measures ANOVA, F = 22.81, df = 1, p<0.01; post-hoc Fisher's Least Significant Difference (LSD) test, df = 4.6, p = 0.017; Figure 2A). Moreover, when presented with faces, S/L monkeys spent less total time looking in the eye region than L/L monkeys did (11.9±4.6% for S/L versus 16.9±11.9% for L/L; Repeated measures ANOVA, MS = 0.45, SS = 0.45, F = 15.24, df = 1, p = 0.017; LSD t-test; Figure 2B ). Animals with the two genotypes did not spend significantly different amounts of time observing the mouth region (p>0.3). For both genotypes, there was a main effect of social status of the stimulus monkey, regardless of genotype: both S/L and L/L monkeys looked in the eye region of high-status faces significantly less than that of low-status faces (12.3±8.2% for high status images versus 16.4±9.5% for low status images; Repeated measures ANOVA, F = 20.45, df = 1, p = 0.011). In contrast, mean pupil diameter, normalized across the experimental session for each monkey, was larger in S/L monkeys when looking at pictures of high-status faces than low-status faces (1.02±0.03 vs. 0.98±0.02), a difference that was absent in L/L monkeys. (0.98±0.02 vs. 0.99±0.02; Repeated measures ANOVA, F = 7.743, df = 1, p = 0.05; post hoc t-test df = 6.76, p = 0.028; Figure 2C). Because of the small sample size, significant results reported here were also analyzed using non-parametric statistics, yielding similar results (Figure S1).

### 5-HTTLPR genotype modulates social influences on risk-taking

In the second experiment, we tested eight adult male macaques (ages 4–10 years, four L/L and four S/L) on a simple economic risk sensitivity assay [12], [13]. Seven of these monkeys were the same as those tested in the free viewing task described above (the 8^th^ was unavailable for study due to training constraints). Monkeys chose between two options, one yielding a fixed-volume juice reward (“safe” option) and the other yielding either a larger or smaller reward with 50% probability of each (“risky” option; Figure 1C). Prior to each choice, the monkeys were primed with a brief (500 ms) presentation of an image belonging to one of the four image pools used in the previous experiment (high status male faces, low status male faces, female perinea, or gray square).

**Figure 3:**
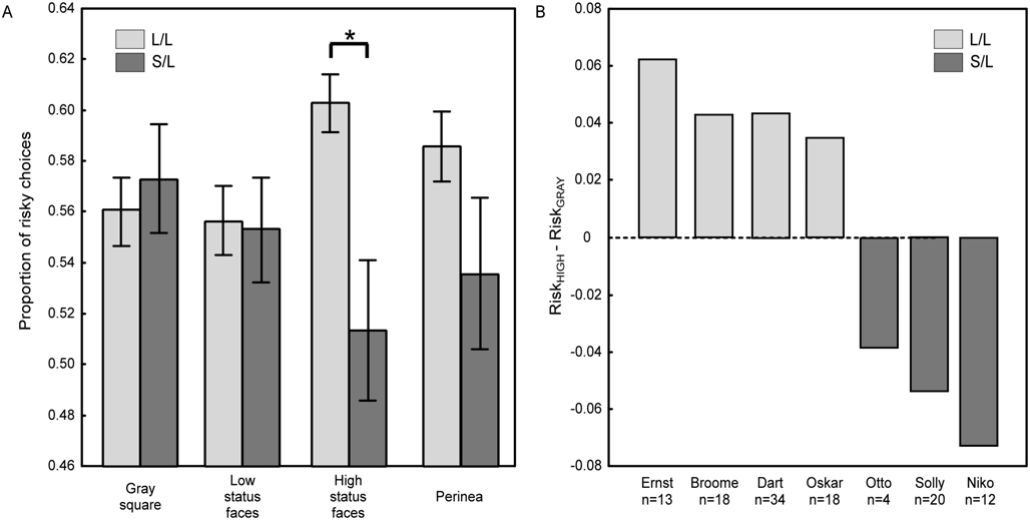
Serotonin transporter genotype influences socially primed risk-sensitivity. (A) Preference for the risky option was suppressed in S/L macaques when primed with a dominant face (* indicates p<0.001). Choices did not differ in L/L and S/L animals when primed with a gray square, subordinate face, or perinea. (B) Each individual L/L subject in the study showed an increased preference for the risky option, and each individual S/L subject showed a decreased preference for the risky option, when primed with a high status face versus a gray square.

Consistent with previous reports [12], [13], monkeys were risk seeking overall (percent risky choice 57.0±0.13, p = 10^−6^, t = 11.84, df = 482, t-test of means against neutral risk preference). Overall, there was a significant main effect of genotype on the propensity to gamble (factorial ANOVA across experimental sessions, F = 6.55, df = 1, p = 0.006; percent risky for L/L 57.7±11.9; percent risky for S/L 54.5±15.5). However, there was a significant interaction between genotype and image category on risk-seeking (F = 3.251, df = 3, p = 0.012), in absence of a main effect for image category (F = .150, df = 3, p = 0.93). Post-hoc t-tests revealed that S/L monkeys chose the risky option significantly less often than the L/L monkeys did when primed with an image of a high status male face (LSD t-test, df = 475, p = 0.0005), whereas there were no significant differences between S/L and L/L animals for the other three image categories ((gray square, p = 0.54; subordinate face, p = 0.53, perinea, p = 0.24; Figure 3A). Because of the small sample size, we also analyzed the data using non-parametric statistics, which yielded similar results (Figure S1).

As an additional measure, we calculated a risk priming by status index, RISKDIFF_high_, consisting of each individual monkey's mean proportion of choosing the risky option when primed with the high-status images minus the mean proportion of choosing the risky option when primed with the gray square. A t-test comparing RISKDIFF_high_ revealed a highly significant difference between the two genotypes (5.0±1.3% for L/L versus −5.0±2.2% for S/L; t = 7.69, df = 5, p = 0.0006; Figure 3B). The equivalent measures for low status faces and perinea were not significantly different between genotypes (p = 0.21 and p = 0.15, respectively).

### 5-HTTLPR genotype modulates economic payout for social images in rhesus macaques

In the final experiment, we used a “pay-per-view” paradigm in order to obtain an implicit measure of the reinforcing value of the social images used in the previously described studies. Due to the length of study needed to perform this assessment, only two adult male rhesus macaques with the L/L genotype and two with the S/L genotype were tested (one high status and one low status individual in each genotype group). Monkeys chose between a juice reward paired with the brief presentation of an image and a juice reward delivered without any accompanying visual stimuli (Figure 1D). The volume of the juice reward and the type of image varied in blocks. This design allowed us to calculate the reinforcing value of each image category in a fluid currency [10], [14]. Images belonged to one of four types: high status male faces, low status male faces, female perinea, or a gray square (Figure 1A).

**Figure 4:**
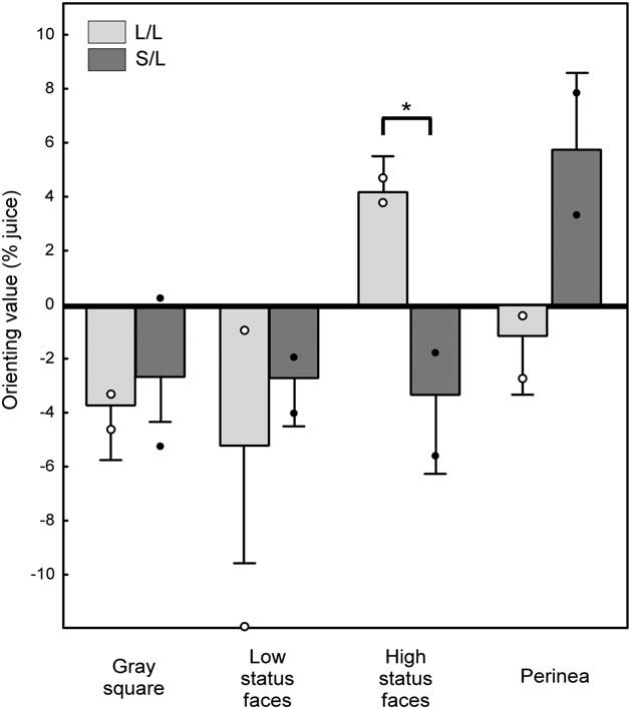
Serotonin transporter genotype modulates social reinforcement in a pay-per-view task. L/L animals sacrificed juice to see high status faces, while S/L monkeys required overpayment to view the same images. Circles indicate mean orienting value for each individual subject.

The point of subjective equality (PSE, see methods) was estimated for each image category for every experiment; the sign-inverted PSE served as a measure of image value [cf. 14]. There was a significant interaction between image category and genotype on image value (factorial ANOVA across experimental sessions, F = 2.95, df = 3, p = 0.035; Figure 4), as well as a main effect of image category (F = 2.67, df = 3, p = 0.049), but there was no main effect of genotype (F = 0.169, df = 1, p = 0.68). L/L monkeys tended to sacrifice fluid to see images of high status males (mean payment amount 2.9±4.2%, t-test of single means against zero, t = 3.30, p = 0.003), whereas S/L monkeys were indifferent or required fluid payment to view the same images (mean payment amount −2.2±8.7%, t-test of single means against zero, t = −1.17, p = 0.25). Post-hoc Fisher's Least Significant Difference (LSD) t-tests (df = 156) revealed that L/L animals paid more than S/L animals to view a high status face (p = 0.028). We also observed a trend towards S/L animals paying more than L/L animals to view images of female perinea (p = 0.066), but there were no significant differences between the two genotype groups in the amount of juice sacrificed for the gray square (p = 0.78) or low status face (p = 0.50) categories. T-tests indicated that the value of the gray square was not significantly different from zero for either group (p = 0.059 and p = 0.12 for L/L and S/L, respectively), thus ruling out the possibility that genotype effects on social reactivity reflected differential sensitivity to fluid reward.

Because of the small sample size, we additionally performed non-parametric tests on the data, consisting of chi-squared analyses comparing the frequency across all sessions that animals with each genotype preferred a particular image category (as measured by PSE) more than the session average. According to this measure, L/L monkeys preferred the high-status faces over the daily average 87% of the time, whereas S/L monkeys only preferred high-status faces more than the daily average 41% of the time (chi-square = 10.41, p = 0.0013, df = 1). Chi-squared tests of the analogous measurements for the gray square, low-status faces, and perinea revealed no significant differences between genotypes (p = 0.45, 0.28, and 0.87, respectively).

### Additional genotypes do not predict social reward and punishment in rhesus macaques

Although we focused our analysis on the length variation in 5-HTTLPR, we were concerned that the results might reflect linkage disequilibrium in a potentially inbred subject population. We note that the macaques used in this study were obtained from three different breeding facilities, and that available pedigree information indicates animals obtained from the same colony are unrelated (Figure S2).

In order to further address the concern of linkage disequilibrium, we performed a retrospective analysis of the behavioral data using additional genotype information. We included analysis of four different single nucleotide polymorphisms (SNPs) related to the gene encoding tryptophan hydroxylase 2 (TPH2) and of a repeat sequence in the upstream regulatory region of the monoamine oxidase A (MAOA) (Figure S3). Like 5-HTTLPR, both TPH-2 and MAOA influence serotonergic function. In each case, either there was no genetic variation in our population or individual differences in social reward and punishment in our tasks did not vary with genotype (Figure S3). Finally, we also genotyped our colony for three SNPs located within the 3′ untranslated region of the serotonin transporter gene. These SNPs had either no explanatory power for the results of our experiments, or lacked sufficient variation to test their effects on the experimental outcomes (Figure S3). These control analyses give us more confidence that variation in the 5-HTTLPR gene contributes to social reward and punishment in rhesus macaques, although this conclusion clearly merits further study with larger populations of rhesus macaques.

## Discussion

Our results endorse the notion that the short 5-HTTLPR allele confers enhanced aversion to social threats. We found that rhesus monkeys carrying the short allele (S/L) were less likely than monkeys homozygous for the long allele (L/L) to gaze directly at the faces and eyes of conspecifics, and that they exhibit a larger sympathetic response, as measured by pupil dilation, to images of high status individuals. Furthermore, we found that 5-HTTLPR genotype was differentially associated with socially primed risk-taking behavior: compared to L/L animals, S allele carriers were significantly less likely to take a gamble after seeing a high status face, whereas risk preferences did not differ between the two groups when gambles were preceded by non-threatening stimuli such as low status faces or reproductive images. Third, in a direct economic measure of how much animals with the two genotypes value various types of social images, we found that S/L monkeys will not give up juice in order to see an image of a high status male face. (See Figure S4 for a comparison of current results with a prior study using the same task [14]). Importantly, the results from these three experiments suggest that 5-HTTLPR genotype influences both social appraisal and nonsocial decision making when it occurs in a social context.

For both human and non-human primates, faces and eyes are a rich source of social information, and allow the observer to gauge the identity, affect, and intention of another individual [15]–[17]. It is well established that both humans and in rhesus macaques tend to preferentially direct visual attention towards the faces of others, especially the eye region [18]–[21]. This visual bias emerges at around 2 months of age in the typically developing human [22], and it is known to be attenuated or absent in individuals with autism and social phobia [23]–[26]. This information comes at a cost, however: one of the functions of eye contract is to establish social dominance [27], and the return of direct eye contact may be construed as a social challenge that is likely to invite retaliation, especially from high-ranking males [28], [29]. The reluctance of S/L macaques to gaze directly at the eyes and faces of conspecifics, as well as their enhanced sympathetic response to the images of high-status males, suggest that S/L macaques experience greater anxiety than L/L macaques when viewing potential social threats.

Given that the S/L monkeys found images of high status male faces more arousing, as measured by pupil diameter, in the free-viewing task, we expected that image-primed gambling would also vary as a function of 5-HTTLPR genotype. In humans, it is well established that mood or disposition can influence risk preferences [30], as well as influence subjective estimates of the frequency of similarly valenced events [31]. According to the “appraisal tendency framework,” these behavioral inconsistencies result from the tendency for subjective emotional influences to bias reward-processing and decision-making, even when those emotions are irrelevant to the decision at hand [32], [33]. These biases are not strictly limited by the valence of emotion. For example, fear-evoking stimuli induce risk aversion whereas anger-eliciting stimuli induce risk seeking in human subjects [33]. In our experiment, preference for a risky outcome over a safe outcome was diminished in S/L monkeys when the decision was preceded by presentation of a potentially threatening image, consistent with the idea that faces of high status males elicit greater fear in S/L monkeys than L/L monkeys.

In the third experiment, we demonstrated that rhesus monkeys heterozygous for the long and short allele (S/L) were less likely than monkeys homozygous for the long allele (L/L) to give up juice in order to see an image of a dominant male face. In the case of the S/L animals, the avoidance of dominant male faces likely results from heightened anxiety associated with this particular category of images. This interpretation is consistent with neuroimaging studies that show that human S carriers have greater activation of the amygdala than L/L homozygotes when looking at angry faces [7], [8], as well as our finding of increased pupil diameter in rhesus S carriers in response to dominant male faces. Within the context of rhesus macaque social structure, this makes adaptive sense: high ranking male macaques are potent social threats, and direct eye contact is a social challenge that is likely to invite retaliation [28], [29].

Differential behavior of S/L and L/L monkeys associated with viewing male faces, particularly those of high status individuals, may reflect heightened anxiety in S allele carriers. This interpretation is motivated by neuroimaging studies showing that S carriers have greater activation of the amygdala, a brain nucleus associated with fear and anxiety, than L/L homozygotes when viewing angry faces [7], [8]. Studies also show that human S carriers are at elevated risk of alcoholism and depression after having experienced childhood abuse or trauma [2], [34]. Such results are consistent with the idea that variation in 5-HTTLPR exerts particular influence during early postnatal development to result in long lasting behavioral changes. Indeed, serotonin plays a critical role in early development by modulating neurogenesis and axonal and dendritic branching [35].

Our findings support the hypothesis that the 5-HT system plays an important role in emotion regulation and social cognition [36], and strengthens the notion that the 5-HTTLPR genotype contributes to disorders associated with social behavior and anxiety. Serotonergic polymorphisms have an additive effect [see 37 for review], and 5-HTTLPR variation may be one of several genotypic factors that contribute to complex disorders such as autism. 5-HTTLPR may also influence the risk for affective and other behavioral disorders through gene-environment interactions.

The commonality of the S allele in both human and rhesus macaque populations, in conjunction with its relative absence in other non-human primate species, suggests that it confers some sort of adaptive advantage [38]. Although the S allele is associated with increased stress and a predisposition to pathological behavior, the fitness advantages associated with heightened social vigilance may well offset these costs. We contend that heightened sensitivity to social threats conferred by the S allele may prove to be adaptive in many contexts, since success in a social group depends on seizing opportunities while simultaneously avoiding potentially harmful antagonistic interactions.

## Materials and Methods

### Subjects and housing

Subjects were 9 adult male rhesus macaques (*Macaca mulatta*) ranging in weight from 7 to 15 kg (mean weight 10.6 kg) and ranging in age from 4 to 10 years (mean age 5.7 years). Macaques were pair housed and had auditory and visual contact with the rest of the colony, consisting of 2 additional males and 4–6 females. The relatedness of the monkeys used in our study is likely to be low, as they were obtained from three different colonies (Figure S2). Pedigree information indicated that two monkeys obtained from a single colony had a coefficient of relatedness of 0.07%, and were thus effectively unrelated [39], [40].

4 of 8 monkeys in the free viewing task were concurrently participating in experiments that required them to fixate on social images. For this reason, training history was included as a categorical predictor in all free viewing analyses.

Monkeys were on controlled fluid access outside of experimental sessions; they earned roughly 80% of their total daily fluid ration during experimental sessions. All testing was conducted in accordance with the PHS Guide to the Care and Use of Laboratory Animals and approved by Duke University Institutional Animal Care and Use Committee.

### Genotyping

5-HTTLPR genotyping was performed by Dr. Robert Ferrell at the University of Pittsburgh Department of Human Genetics, as described previously [41]. Briefly, animals were anesthetized with ketamine (3 mg/kg i.m.) and domitor (0.15 mg/kg i.m.) and peripheral blood was drawn. Genomic DNA was isolated using the PureGene kit following the manufacturers instructions (Gentra Systems) and 5-HTTLPR was amplified using oligonucleotide primers rhMUT 5′-TCG ACT GGC GTT GCC GCT CTG AAT GC-3′and rhINT 5′-CAG GGG AGA TCC TGG GAG GGA-3′.

### Stimuli and Behavioral Paradigms

Procedures were as described in detail elsewhere [14], with the following modifications. Eye metrics in the free viewing and the socially primed gambling experiments were monitored with an infrared camera (Eyelink 1000, SR Research, Osgoode ON) at a rate of 1000 Hz. Both eye position and pupil diameter were recorded in the free viewing experiment. For the gambling and pay-per-view experiments, only eye position was recorded. In the pay-per-view task, eye position measured with the Eyelink system in three of the monkeys and with a scleral search coil system (Riverbend) in one monkey.

Monkeys performed the behavioral tasks seated comfortably in a primate chair with their eyes approximately 40 cm from the computer monitor. Stimuli were presented using Matlab Psychophysics Toolbox extensions (free viewing task [42], [43]), or the Gramalkn Experiment Control System (primed risk and pay-per-view tasks; ryklinsoftware.com) on a Dell Optiplex GX 620 computer.

Social image stimuli used in the free viewing experiment consisted of color bitmaps displayed on either a 1024×768 or 1280×1024 monitor at a 75 Hz refresh rate. Images were scaled so that they were approximately 7.5×8 visual degrees on both monitor sizes. Social images consisted of faces of familiar individuals housed in the same room as the subjects. Some subjects saw images of themselves; however, previous results suggest that subjects respond to images of themselves according to their status [14]. Both high status and low status male pools consisted of 20 face images of 3 different individuals whose social status remained stable over the duration of the experiment. High status and low status scrambled face pools consisted of 18 grid-wise scrambled face images. Each image was shown exactly once per session. For each face image, rectangular regions of interest (ROIs) containing the eyes and mouth were manually defined. No ROIs were defined for the scrambled faces, as anatomical features were indistiguishable in these stimuli.

Social image stimuli used in both the pay-per-view and socially primed gambling experiments consisted of color bitmaps ranging in size from 115×115 to 130×130 pixels displayed on a 1024×768 monitor at a 60 Hz refresh rate. Images were drawn randomly with replacement from one of the following four image pools: high status male, low status male, female perinea, or gray square. All image pools except the gray square consisted of 60 images. Similar to the free viewing experiment, both high status and low status male pools consisted of 20 face images of 3 different individuals whose social status remained stable over the duration of the experiment. The female perinea pool consisted of 60 images from 4 different individuals. The gray square image pool consisted of a single image.

### Free viewing task

Each trial of the free viewing task was initiated with a 400 ms tone, after which the animal was required to fixate on a small visual target whose location was randomly selected from one of nine spatially distributed locations. Monkeys were required to fixate within a 2 degree window of the target for 300 ms and were then rewarded with a short (500 ms) tone and small liquid reward. After a 400 ms pause, a centrally located image was presented for three seconds. Animals were not required to fixate or otherwise look at the image. Intertrial intervals (ITIs) were 400,600 or 800 ms.

### Socially primed gambling task

Socially primed gambling trials were initiated with a 300 ms tone, after which the animal was required to fixate on a central point. After either 350 or 400 ms, the central point was replaced with an image from one of four image pools (see Stimuli) for 500 ms, and then replaced with the fixation point for an additional 350 or 400 ms. Offset of the central fixation point permitted the monkeys to choose one of two targets displayed diametrically around the central point. Choosing the “safe” target delivered a constant amount of juice on every trial, while choosing the “risky” target randomly delivered a juice reward of less or more than the safe amount of juice with probability 0.5. The locations of the safe and risky targets were varied every 20–30 trials, with each block consisting of 40–60 trials counterbalanced for the spatial location of the safe and risky targets. Rewards were delivered after fixation on a target for 200 ms; no reward was given if the monkeys failed to complete the trial. A 300 ms broadband noise preceded juice delivery on all correct trials. In order to encourage sampling of both options, 20% of trials consisted of forced saccade trials towards either the safe or risky target. Trials of this type were randomly interspersed; the remaining 80% of trials were choice trials. Intertrial intervals were fixed at 700 ms.

### Pay-per-view task

The pay-per-view task was performed as described previously [14]. Briefly, monkeys fixated on a central point. After 350 or 400 ms, two identical, eccentric, and diametrically opposed targets (T1 and T2) appeared for 300, 400 or 500 ms, during which the monkey was required to maintain fixation on the central point. The locations of T1 and T2 remained fixed for the duration of the experiment. Offset of the central point cued the animal to choose either T1 or T2 with a gaze shift. After fixating the eccentric target for 200 ms, a 300 ms tone and a juice reward were delivered. If the animal chose T1, there was a 1200 ms ITI; if the animal chose T2, there was a 500 ms presentation of an image followed by a 700 ms ITI. In order to encourage sampling of both options, 20% of trials consisted of forced trials towards either T1 or T2. Trials of this type were randomly interspersed; the remaining 80% of trials were choice trials.

### Statistical analysis

Behavioral data were analyzed off-line using Matlab (Mathworks, Natick, Massachusetts) and Statistica (Statsoft, Tulsa, OK).

For the free viewing task, the percentage of samples that the monkey's eye position fell within the boundaries of the image was calculated for each trial. For trials in which faces were displayed, the percentage of samples that the eye position fell within each of two (eyes, mouth) ROIs were also determined. Mean pupil diameter was determined for each trial. To control for interindividual differences in pupil size and also for session-by-session variance in ambient lighting, pupil diameter on each trial was normalized to the mean diameter as calculated across the entire experimental session. Data were averaged across both experimental sessions for each monkey and repeated measures (RM) ANOVAs were performed, followed by post-hoc Fisher LSD t-tests. Categorical predictors SERT genotype and training level, as well as within subject measures “stimuli status” (ie. High- vs. low- status image pool) and “face vs. scrambled” were included in the analysis of the amount of time spent viewing each picture. For ROI analysis, RM ANOVAs were performed using genotype, training level, and stimuli status as regressors. Analysis of the pupil diameter data consisted of RM ANOVAs directly comparing the effects of high- vs. low status face pictures, and included genotype and training level as predictors.

For the socially primed gambling task, each mean choice frequency of the risky target in each spatially counterbalanced block was considered a single data point. Sidebiases were calculated for each data point by calculating the percent of time the subject chose a single lateralized target for each counterbalanced session. Only data with sidebias measures within two standard deviations of the mean were included for analysis. Choice frequencies were analyzed across experimental sessions using factorial ANOVAs (percent risky×image category×genotype), followed by post-hoc Fisher LSD t-tests.

For the pay-per-view task, the point of subjective equivalence (PSE) was estimated by a cumulative normal function fit to proportion of trials monkeys chose T2 as a function of the difference in juice delivered for T1 and T2 choices [14]. Each PSE comprised a single data point considered in the statistical analysis for the pay-per-view image valuation. A factorial ANOVA (PSE×image category×genotype) was performed to analyze pay-per-view data, followed by post-hoc Fisher LSD t-tests. Image viewing time analyses were considered on a trial-by- trial basis and were again analyzed using factorial ANOVAs (normalized viewing time×image category×genotype).

## Supporting Information

Figure S1 Nonparametric statistics for experiments 1 and 2
(0.02 MB DOC)

Figure S2 Relatedness of subjects
(0.03 MB DOC)

Figure S3 Additional genotyping results
(0.05 MB DOC)

Figure S4 Comparison of current and previously published results in the pay-per-view task
(0.02 MB DOC)

## References

[1] Lesch KP, Bengel D, Heils A, Sabol SZ, Greenberg BD (1996). Association of anxiety-related traits with a polymorphism in the serotonin transporter gene regulatory region.. Science.

[2] Caspi A, Sugden K, Moffitt TE, Taylor A, Craig IW (2003). Influence of life stress on depression: Moderation by a polymorphism in the 5-HTT gene.. Science.

[3] Barr CS, Newman TK, Shannon C, Parker C, Dvoskin RL (2004). Rearing condition and rh5-HTTLPR interact to influence limbic-hypothalamic-pituitary-adrenal axis response to stress in infant macaques.. Biological Psychiatry.

[4] Champoux M, Bennett A, Shannon C, Higley JD, Lesch KP (2002). Serotonin transporter gene polymorphism, differential early rearing, and behavior in rhesus monkey neonates.. Molecular Psychiatry.

[5] Bennett AJ, Lesch KP, Heils A, Long JC, Lorenz JG (2002). Early experience and serotonin transporter gene variation interact to influence primate CNS function.. Molecular Psychiatry.

[6] Barr CS, Newman TK, Becker ML, Champoux M, Lesch KP (2003). Serotonin transporter gene variation is associated with alcohol sensitivity in rhesus macaques exposed to early-life stress.. Alcoholism-Clinical and Experimental Research.

[7] Hariri AR, Mattay VS, Tessitore A, Kolachana B, Fera F (2002). Serotonin transporter genetic variation and the response of the human amygdala.. Science.

[8] Hariri AR, Drabant EM, Munoz KE, Kolachana LS, Mattay VS (2005). A susceptibility gene for affective disorders and the response of the human amygdala.. Archives of General Psychiatry.

[9] Deaner RO, Shepherd SV, Platt ML (2005). Social context influences gaze-following and neuronal activity in macaque area LIP (Abstract).. Journal of Vision.

[10] Klein JT, Deaner RO, Platt ML (2008). Neural correlates of social target value in macaque parietal cortex.. Curr Biol In press.

[11] Loewenstein GF, Weber EU, Hsee CK, Welch N (2001). Risk as feelings.. Psychological Bulletin.

[12] McCoy AN, Platt ML (2005). Risk-sensitive neurons in macaque posterior cingulate cortex.. Nat Neurosci.

[13] Hayden BY, Platt ML (2007). Temporal discounting predicts risk sensitivity in rhesus Macaques.. Current Biology.

[14] Deaner RO, Khera AV, Platt ML (2005). Monkeys pay per view: adaptive valuation of social images by rhesus macaques.. Curr Biol.

[15] Rosenfeld SA, Vanhoesen GW (1979). Face Recognition in the Rhesus-Monkey.. Neuropsychologia.

[16] Emery NJ (2000). The eyes have it: The neuroethology, function and evolution of social gaze.. Neuroscience & Biobehavioral Reviews.

[17] Mendelson MJ, Haith MM, Goldmanrakic PS (1982). Face Scanning and Responsiveness to Social Cues in Infant Rhesus-Monkeys.. Developmental Psychology.

[18] Ghazanfar AA, Nielsen K, Logothetis NK (2006). Eye movements of monkey observers viewing vocalizing conspecifics.. Cognition.

[19] Yarbus AL (1967). Eye Movements and Vision..

[20] Nahm FKD, Perret A, Amaral DG, Albright TD (1997). How do monkeys look at faces?. Journal of Cognitive Neuroscience.

[21] Guo K, Robertson RG, Mahmoodi S, Tadmor Y, Young MP (2003). How do monkeys view faces?-a study of eye movements.. Experimental Brain Research.

[22] Maurer D, Salapatek P (1976). Developmental-Changes in Scanning of Faces by Young Infants.. Child Development.

[23] Spezio ML, Adolphs R, Hurley RSE, Piven J (2007). Analysis of face gaze in autism using “Bubbles”.. Neuropsychologia.

[24] Klin A, Jones W, Schultz R, Volkmar F, Cohen D (2002). Visual Fixation Patterns During Viewing of Naturalistic Social Situations as Predictors of Social Competence in Individuals With Autism.. Arch Gen Psychiatry%R 101001/archpsyc599809.

[25] Pelphrey KA, Sasson NJ, Reznick JS, Paul G, Goldman BD (2002). Visual scanning of faces in autism.. Journal of Autism & Developmental Disorders.

[26] Horley K, Williams LM, Gonsalvez C, Gordon E (2003). Social phobics do not see eye to eye: A visual scanpath study of emotional expression processing.. Journal of Anxiety Disorders.

[27] Kleinke CL (1986). Gaze and Eye Contact-a Research Review.. Psychological Bulletin.

[28] van Hoof JARAM, Morris D (1967). The facial displays for the catarrhine monkeys and apes.. Primate Ethology.

[29] Hauser MD (1996). The Evolution of Communication..

[30] Tversky A, Kahneman D (1981). The Framing of Decisions and the Psychology of Choice.. Science.

[31] Johnson EJ, Tversky A (1983). Affect, Generalization, and the Perception of Risk.. Journal of Personality and Social Psychology.

[32] Lerner JS, Tiedens LZ (2006). Portrait of the angry decision maker: How appraisal tendencies shape anger's influence on cognition.. Journal of Behavioral Decision Making.

[33] Lerner JS, Keltner D (2000). Beyond valence: Toward a model of emotion-specific influences on judgement and choice.. Cognition & Emotion.

[34] Uher R, McGuffin P (2008). The moderation by the serotonin transporter gene of environmental adversity in the aetiology of mental illness: review and methodological analysis.. Molecular Psychiatry.

[35] Gaspar P, Cases O, Maroteaux L (2003). The developmental role of serotonin: News from mouse molecular genetics.. Nature Reviews Neuroscience.

[36] Canli T, Lesch KP (2007). Long story short: the serotonin transporter in emotion regulation and social cognition.. Nature Neuroscience.

[37] Murphy DL, Lesch KP (2008). Targeting the murine serotonin transporter: insights into human neurobiology.. Nature Reviews Neuroscience.

[38] Suomi SJ (2006). Risk, resilience, and gene×environment interactions in rhesus monkeys.. Resilience in Children.

[39] Bellamy RJ, Inglehearn CF, Jalili IK, Jeffreys AJ, Bhattacharya SS (1991). Increased Band Sharing in DNA Fingerprints of an Inbred Human-Population.. Human Genetics.

[40] Wright S (1922). Coefficients of Inbreeding and Relationship. The University of Chicago Press for The American Society of Naturalists..

[41] Wendland JR, Lesch KP, Newman TK, Timme A, Gachot-Neveu H (2006). Differential functional variability of serotonin transporter and monoamine oxidase a genes in macaque species displaying contrasting levels of aggression-related behavior.. Behavior Genetics.

[42] Brainard D (1997). The Psychophysics Toolbox.. Spatial Vision.

[43] Pelli DG (1997). The VideoToolbox software for visual psychophysics: Transforming numbers into movies.. Spatial Vision.

