# Supplementary material for: Serotonin Transporter Genotype Modulates Social Reward and Punishment in Rhesus Macaques
Source: PLoS One. 2009 Jan 14;4(1):e4156. doi: 10.1371/journal.pone.0004156 (PMC2612746; doi:10.1371/journal.pone.0004156)
Supplement: Figure S1 — Nonparametric statistics for experiments 1 and 2 (0.02 MB DOC) [file pone.0004156.s001.doc]

Non parametric statistics

1. Experiment 1- Social image viewing

Across all trials, the percentage of time spent viewing each image varied according to genotype for face images (Mann-Whitney U test, Z=8.89, p<10^-6^) but not for scrambled faces (Z=-1.76, p=0.08). S/L monkeys looked at the eye region of face images for significantly less time than L/L monkeys (Mann-Whitney U test, Z=3.40, p=0.0007), and had larger normalized pupil diameters when looking at high status faces (Mann-Whitney U test, Z=3.13, p=0.002) but not low status faces (Z=-0.28, p=0.78).

1. Experiment 2 - Socially primed gambling

Individual Mann-Whitney U tests were performed for each image category across all experimental blocks, using the percentage of trials the monkey chose to gamble as the dependent variable and 5-HTTLPR genotype as the categorical predictor. L/L monkeys gambled significantly more during high-status image blocks than did S/L monkeys (Z=2.94, p=0.003). There were no significant differences in gambling behavior for the other three image categories, although a trend towards significance was noted in the case of perinea images (gray square, p= 0.53; low-status faces, p=0.98; perinea, p=0.07).
