# Supplementary material for: Serotonin Transporter Genotype Modulates Social Reward and Punishment in Rhesus Macaques
Source: PLoS One. 2009 Jan 14;4(1):e4156. doi: 10.1371/journal.pone.0004156 (PMC2612746; doi:10.1371/journal.pone.0004156)
Supplement: Figure S2 — Relatedness of subjects (0.03 MB DOC) [file pone.0004156.s002.doc]

**Supplementary Information: Relatedness of subjects**

The relatedness of the 8 monkeys used in our study is likely to be low, as they were obtained from three different colonies (Table 1). Extended pedigrees were available for animals obtained from colony A; based on this information, we determined Wright’s coefficient of relatedness to be 0.07%, indicating that the two individuals, despite being from the same colony, were effectively unrelated [1,2]. Although pedigree information was lacking for animals obtained from colony B, we confirmed that each individual had a different sire and dam. Information for animals from colony C could not be obtained.

| Subject name | 5-HTTLPR genotype | Experiment | colony |
| --- | --- | --- | --- |
| Ernst | L/L | I, II, III | A |
| Broome | L/L | I, II, III | B |
| Dart | L/L | I, II | B |
| Harry | L/L | I | A |
| Oskar | L/L | II | A |
| Solly | L/S | I, II | C |
| Sherry | L/S | I, III | C |
| Niko | L/S | I, II, III | B |
| Otto | L/S | I, II | B |

Table 1. Subjects were derived from three different breeding colonies. Experiment I = free viewing; II = primed risk; III = pay-per-view.

1. Bellamy RJ, Inglehearn CF, Jalili IK, Jeffreys AJ, Bhattacharya SS (1991) Increased Band Sharing in DNA Fingerprints of an Inbred Human-Population. Human Genetics 87: 341-347.

2. Wright S (1922) Coefficients of Inbreeding and Relationship. The University of Chicago Press for The American Society of Naturalists. pp. 330-338.
