# Supplementary material for: Serotonin Transporter Genotype Modulates Social Reward and Punishment in Rhesus Macaques
Source: PLoS One. 2009 Jan 14;4(1):e4156. doi: 10.1371/journal.pone.0004156 (PMC2612746; doi:10.1371/journal.pone.0004156)
Supplement: Figure S3 — Additional genotyping results (0.05 MB DOC) [file pone.0004156.s003.doc]

**Supplementary information: Additional genotyping results**

**Monoamine oxidase A (*MAOA*) gene**

*MAOA* selectively degrades serotonin, norephinephrine, and dopamine following reuptake from the synaptic cleft. A polymorphism (MAOA-LPR) in the upstream regulatory region of the human MAOA consisting of a variable number of tandem repeats (VNTR) has been shown to affect transcriptional activity in vitro [3, 4] (Deckert et al. 1999; Sabol et al. 1998). This MAOA polymorphism is associated with variability in aggression, and, like 5-HTTLPR, may interact with early environment in order to increase risk of pathological behavior later in life, including antisocial personality and violent criminality [5, 6]. We genotyped our animals for the orthologous rhesus MAOA linked polymorphic region, comprised of a 18-bp sequence with length variation of 5-7 repeats [7](Table 2). As in humans, the rhesus MAOA VNTR interacts with early experience to predict aggression levels in adulthood [8]. Three of eight animals used in this study possessed the 7-repeat allele, which confers low activity (associated with increased aggression in peer-reared ani animals), and the other six possessed the 6-repeat allele, which confers high activity. Because the MAOA gene is located on the X chromosome, homo- versus heterozygosity is not a consideration in our all-male colony.

In the free viewing task, MAOA-LPR had no significant effect on pupil diameter, although the identity of the genotype did interact with training history (see experimental procedures for definition) and picture identity to produce significant effects in picture and eye viewing time. We used a forward entry general regression model to gauge the relative contributions of each predictor on the picture viewing times (predictors: picture status, picture type (face vs. scrambled), training level and genotype; p value criteria for inclusion = 0.15), we found no effects of MAOA genotype once the effects of training were removed (all p> 0.3).  In contrast, a genotype x picture type interaction remained for 5-HTTPLR after the effects of training were removed (p=0.020). We similarly used a forward entry model to analyze the amount of time spent in the eye region (categorical predictors: subject training, picture status, and genotype, p value criteria for inclusion = 0.15), and found no effects of MAOA (all p>0.6) once the effects of training were removed.  In contrast, the analogous model using 5-HTTLPR as the genotype of interest revealed that 5-HTTLPR genotype was the strong predictor of eye viewing (p=0.001) after the effects of training were removed.

In further contrast to the effects of 5-HTTLPR genotype, the MAOA-LPR genotype had no significant interaction with image category in either the pay-per-view task (image category x genotype interaction, df=3, F=1.113, p=0.346) nor the primed risk task (image category x genotype interaction, df=3, F=1.745, p=0.157). There was a significant main effect of MAOA-LPR genotype on the probability of choosing the risky option (df=1, F=12.41, p=0.0005), with the low transcription allele (6 repeats) having significantly fewer risky choices than the high transcription allele (7 repeat) carriers (probability of risky choice for low allele carriers 55.0 ± 13.2 %; for high allele carriers 59.3 ± 12.6% ).

**Tryptophan hydroxylase-2**

The tryptophan hydroxylase-2 (TPH2) isoform codes for the rate limiting enzyme in the neuronal synthesis of serotonin. The identities of four TPH-2 SNPs were genotyped for each member of our subject pool, as performed in Chen, et al [9].

TPH2 -363T>G

Polymorphism TPH2 -363T>G is located upstream of the promoter region and orthologous to human allele -844G>T. This SNP was of particular interest because it appears to have a functional role similar to that of 5-HTTLPR: In humans, carriers of the T variant show greater amygdala activity to emotional compared to neutral faces [10, 11]. More recent studies suggest that TPH2 and 5-HTTLPR gene variants have an additive effect, with individuals carrying both the short variant of the 5-HTTLPR in addition to the T variant of the TPH2 SNP showing the greatest changes in activity in the putamen in response to emotional stimuli [12]. However, all of our macaque subjects in the free viewing and pay per view paradigms, and all but one of our macaque subjects in the risk paradigm are T carriers, which precluded a differentiation of behavior based on this gene variant (Table 2).

TPH2 223G>A

There were no significant effects of genotype on free viewing behavior. Lack of variation in our subject population precluded statistical analysis of the effects of genotype on behavior in the primed risk and pay-per-view tasks. (Table 2).

TPH2 1503 A>G

There was not enough variation in the subject population for statistical analysis in any of the three tasks (Table 2).

TPH2 2051 A>C

The SNP TPH2 2051 A>C is located in the 3’-UTR region and is associated with variations in hypothalamic-pituitary-adrenal axis function [9]. Two out of the seven animals in the risk study are C carriers, which has been shown to be correlated with higher plasma cortisol levels in the morning [9] (Table 2). There was not enough variation in the subject population for statistical analysis of the pay-per-view task, and there were no significant effects of genotype on free viewing behavior. For the socially primed risk task, presence or absence of the C nucleotide at this point did not interact significantly with image category (df=3, F= 0.828, p=0.48) on the tendency to choose the risky option, though there was a main effect of genotype on risk proclivity (df=1, F=4.28, p=0.039).

**3’ UTR of the serotonin transporter gene**

Finally, our colony was genotyped for three SNPs located within the 3’ untranslated region of the serotonin transporter gene (Table 2). Although there was not sufficient variation to test the effects of these SNP identities on the pay-per-view data, dichotomization of the primed risk subjects based on each of the three SNP identities revealed no significant interaction effects between genotype and image category on risk proclivity (p>0.05). For two of the three SNPs (rs45436079 and rs45436078), there was not enough variation to test the effects of genotype on behavior in the free viewing while retaining training history as a predictor. For SNP rs45436083, there were no significant effects of genotype on image-relevant free viewing behaviors.

|  | MAOA | TPH2  -363T>G | TPH2  223G>A | TPH2  1503 A>G | TPH2 2051 A>C | SERT 3’ UTR  rs45436083 | SERT 3’ UTR  rs45436079 | SERT 3’ UTR  rs45436078 |
| --- | --- | --- | --- | --- | --- | --- | --- | --- |
| Ernst | 7/7 | G/T | G/G | A/A | A/C | T/T | C/C | T/T |
| Broome | 6/6 | G/T | G/G | A/A | A/A | T/C | C/G | T/C |
| Dart | 6/6 | G/T | G/G | A/A | A/A | T/T | C/C | C/C |
| Harry | 6/6 | G/T | A/G | A/A | C/C | T/T | C/C | T/T |
| Oskar | 7/7 | G/G | Na | A/A | C/C | C/C | G/G | C/C |
| Solly | 6/6 | T/T | Na | A/G | A/A | T/T | C/G | C/C |
| Sherry | 7/7 | T/T | G/G | A/A | A/A | T/T | C/C | C/C |
| Niko | 6/6 | T/T | G/G | A/A | A/A | T/C | C/C | C/C |
| Otto | 6/6 | T/T | A/G | A/A | A/A | T/C | C/C | C/C |

Table 2. Genotyping results for polymorphisms in three different genes related to the serotonergic system. Rows highlighted in gray indicate L/L animals; rows in yellow indicate S/L animals. MAOA, monoamine oxidase A; TPH2, tryptophan hyroxylase-2; SERT, serotonin transporter; UTR, untranslated region; Na = No answer (genotype could not be obtained).
