# Supplementary material for: Serotonin Transporter Genotype Modulates Social Reward and Punishment in Rhesus Macaques
Source: PLoS One. 2009 Jan 14;4(1):e4156. doi: 10.1371/journal.pone.0004156 (PMC2612746; doi:10.1371/journal.pone.0004156)
Supplement: Figure S4 — Comparison of current and previously published results in the pay-per-view task (0.02 MB DOC) [file pone.0004156.s004.doc]

**Supplementary information: Comparison of current and previously published results in the pay-per-view task**

Although a previous report suggested that “typical” male rhesus macaques sacrifice juice to see dominant male faces as well as female perinea [1], the subjects in that study were not segregated according to 5-HTTLPR genotype, which explains the difference in the results of the two studies. A post-hoc analysis of the former study is precluded by the fact that genotype information cannot be obtained for one of the subjects. We note that, in contrast to the Deaner et al study, one relative weakness of the current study is the difference in training histories across subjects. In the current study, one of the two L/L macaques was newly trained and naïve to the pay-per-view paradigm at the outset of the experiment, whereas the second L/L macaque and both of the S/L macaques had had extensive previous experience with the paradigm. Although new images were incorporated into the stimulus set for this experiment, the individuals represented and the format of the images overlapped with those used in previous experiments this paradigm. Because the pay-per-view paradigm explicitly measures choice preference of the visual stimuli, over-exposure to the stimuli and differences in training histories could be a concern. These concerns are addressed with the addition of a novel primed risk experiment, to which all subjects were naïve.

1. Deaner RO, Khera AV, Platt ML (2005) Monkeys pay per view: adaptive valuation of social images by rhesus macaques. Curr Biol 15: 543-548.
